# SARS-CoV-2 Systemic Effects: New Clues

**DOI:** 10.3390/microorganisms11051209

**Published:** 2023-05-05

**Authors:** Silvia Beltrami, Sabrina Rizzo, Francesca Caccuri, Roberta Rizzo, Daria Bortolotti, Giovanna Schiuma

**Affiliations:** 1 Department of Chemical, Pharmaceutical and Agricultural Science, University of Ferrara, 44121 Ferrara, Italy; 2 Department of Microbiology and Virology, “Spedali Civili”, 25126 Brescia, Italy

To date, much discussion has been had on severe acute respiratory syndrome coronavirus 2 (SARS-CoV-2) lung infection associated with COVID-19 onset, of which the major manifestation is characterized by a “cytokine storm” [1] and acute respiratory distress syndrome (ARDS) in severely affected patients (Figure 1).

ARDS reflects dramatic microvascular endothelial cell (mEC) dysfunction, which encompasses changes in vascular permeability, inflammation, activation of procoagulant pathways and disruption of the alveolar–capillary barrier [2,3] (Figure 1). The study conducted by Caccuri et al. confirms that the SARS-CoV-2 infection of human lung microvascular ECs (HL-mECs) sustains inflammatory and vascular dysfunction, leading to vascular detriment and leakage [4]. Having uncovered the intracellular expression of viral RNA and proteins in the absence of cytopathic effects and infectious viral progeny release, researchers have been able to demonstrate that HL-mECs support an abortive SARS-CoV-2 replication. This occurs without the presence of ACE2 expression, which is necessary for the active replication of SARS-CoV-2 in ECs [5]. 

This observation is implicit of the ability of SARS-CoV-2 to employ an alternative receptor to infect HL-mECs, even though a low-level expression of ACE2 cannot be completely disregarded. Many viruses have an arginine-glycine-aspartic acid (RGD) motif on the viral envelope, recognized by integrins, that is critical in the mechanisms behind virus infection and cell internalization [6,7]. In particular, it was reported that the conserved RGD motif may be a mechanism by which SARS-CoV-2 interacts with integrins. Microarray analysis revealed that following infection, HL-mECs release many pro-inflammatory and pro-angiogenic molecules, which induces the development of an angiogenic phenotype in HL-mECs. The modification of SARS-CoV-2-infected HL-mECs to inflammatory and angiogenic responses was validated by proteome analysis, which also unveiled the expression of antiviral molecules, among which are annexin A6 and MX1.

Considering that ARDS represents one of the major causes of mortality for severe COVID-19 subjects, a therapy based on pulmonary rehabilitation (PR), known to be effective against multiple pulmonary diseases [8,9], has been exploited for COVID-19 treatments [10]. The primary benefits of PR involve the improvement of physical performance, quantified as the functional independence measurement (FIM) and 6 min walking distance (6-MWD), and the wellbeing of patients, described using the feeling thermometer (FT) parameter. However, since not all patients benefit from the PR treatment to the same extent, such as some post-COVID-19 patients, further studies are necessary to identify the reasons for this difference in response to therapy in order to develop optimized concepts within PR [11]. 

Although the respiratory tract represents the main site of entry for the virus, the spectrum of the clinical manifestation of SARS-CoV-2 is wide, since the primary infection could lead to important systemic effects [7].

Theoretically, SARS-CoV-2 can directly invade any organ system that expresses the ACE2 receptor, resulting in symptoms that are vague or unusual [12]. In fact, as the pandemic spread and new SARS-CoV-2 variants arose, more COVID-19 patients experienced several nonspecific or unusual extra-pulmonary symptoms involving different body systems, including systemic inflammation, hypercoagulability and renin–angiotensin–aldosterone system dysregulation (RAAS) [13]. In particular, SARS-CoV-2 infection has been described in association to renal complication, including nephropathies associated with systemic SARS-CoV-2 infection, rhabdomyolysis-associated tubular toxicity and cardiorenal syndrome (such as renal hypoperfusion, hypotension, nephrotoxic drug interactions and venous congestion) [14,15] (Figure 1). In fact, SARS-CoV-2 is able to modulate ACE2 expression in several cells of the cardiovascular system, such as cardiomyocytes, fibroblast and pericytes, triggering the neurohumoral system and resulting in defective contractibility, among other significant cardiac morbidities [16,17,18] (Figure 1). 

In addition, another major site of extrapulmonary infection of SARS-CoV-2 is represented by the gastrointestinal tract, due to the high expression of ACE2 in enterocytes. The incipient manifestations of COVID-induced gastrointestinal (GI) problems include vomiting, diarrhea, abdominal pain, bleeding, diminished appetite or a combination of the former [19]. 

Another important aspect of the core of SARS-CoV-2 gastrointestinal infection is the presence of several comorbidities in patients. Diabetes mellitus is among the most frequently occurring of the major COVID-19 comorbidities [20,21,22,23], often associated to a high risk of severe prognosis [24,25,26]. In fact, the excessive amounts of insulin produced by diabetic patients seem to induce the PI3K/Akt/mTOR pathway, already active in COVID-19, which promotes the release of tumor necrosis factor (TNF) and interleukin-6 (IL-6) [27,28,29], consequently aggravating the inflammatory status already altered in COVID-19 patients [30]. Similarly to observations in pulmonary pathology, some therapeutic strategies could exploit the treatment of certain comorbidities to improve the conditions of COVID-19 patients, such as diabetes mellitus [20,21,22]. Among the main antidiabetic therapies, metformin is one of the most used, consisting of an oral hypoglycemic agent inhibiting the PI3K/Akt/mTOR pathway [31,32,33] that causes inflammation in both diabetes mellitus and COVID-19. For this reason, the use of metformin can be considered a potential anti-inflammatory treatment to improve the prognosis of patients with both COVID-19 and diabetes [34,35].

Nevertheless, besides the several therapeutic approaches, it has been demonstrated that prevention is critical in decreasing infection rates and sequelae. Although vitamin D3 supplementation is still controversial in the prevention of infection [36,37], a meta-analysis asserted that a low serum 25-hydroxyvitamin D3 [25(OH)D3] level was associated with a higher risk of SARS-CoV-2 infection [38]. Similarly, Romero-Ibarguengoitia et al. [39] showed that individuals with 25(OH)D3 levels between 20 and 100 ng/mL and vitamin D3 supplementation have a lower rate of SARS-CoV-2 infection, reinforcing the importance of supplementation in the prevention of COVID-19.

The importance of therapies and prevention appears to be crucial in view of the ability of SARS-CoV-2 to infect a wide range of tissues and organs. Recently, even more interest has been paid to SARS-CoV-2 infection at the reproductive tract level. In particular, the male reproductive system could present peculiar clinical manifestations in response to SARS-CoV-2 infection, possibly leading to exacerbated conditions due to a stronger type I immune response, characterized by a lower CD4/CD8 T cell ratio [40]. The increased ACE2 expression, and the levels of transmembrane protease serine 2 (TMPRSS2) and cathepsins [41,42] within the testes, and the deleterious role of testosterone in the interim of infection, could impede spermatogenesis and cause male infertility [43] (Figure 1). Despite SARS-CoV-2 infection potentially resulting in testicular damage and testosterone level impairment, whether these consequences of certain severe COVID-19 cases is caused by direct SARS-CoV-2 infection, indirect inflammatory and oxidative stress, or a combination of these mechanisms, is not completely clear. The study conducted by Campos et al. suggested that testicular damage observed in severe COVID-19 cases could be partly due to a direct SARS-CoV-2 infection of testicular cells. In fact, in a study conducted in an animal model, SARS-CoV-2 RNA was detected in the testes of golden Syrian hamsters infected intranasally, which also showed signs of mild disease. Most of the viral RNA was found during the first week following infection, without any significant histopathological damage. Moreover, the hamster testes exposed to SARS-CoV-2 ex vivo were susceptible to infection, as demonstrated by increasing virus titers in the medium and the presence of viral RNA in the seminiferous tubules and the interstitium. In contrast, SARS-CoV-2 titers remained stable in hpSertoli cells, suggesting that these cells might support low levels of SARS-CoV-2 infection [44].

Despite the female reproductive tract expressing low ACE2 levels than testes, SARS-CoV-2 infection seriously considered within fertility clinics, as the infection has the potential to be implicated in placental annexes. Because of the peculiar tolerogenic environment needed to protect the semi-allogenic fetus from the maternal immune system attack during pregnancy, a dysregulated inflammatory response to viruses may occur, probably due to a defective interferon response known to be crucial in antiviral responses [45]. In fact, in normal pregnancy and immunocompetent physiological conditions, IFN-γ plays a pivotal immunomodulatory role [46], thus it might be supposed that SARS-CoV-2 infection could affect the pregnancy course by specifically modulating IFN-γ levels. This hypothesis is supported by Cennamo et al., who observed significantly lower IFN-γ amounts in the peripheral and cord blood of pregnant COVID-19-infected women (Figure 1), suggesting that this alteration, possibly due to SARS-CoV-2 infection as an attempt to subvert the IFN-γ antiviral effect, could affect the fetal microenvironment, increasing the viral susceptibility of newborns [47].

This evidence confirmed the importance of a correct activation of innate immune response to efficiently counteract SARS-CoV-2 and infection susceptibility. Nevertheless, the cytokine storm condition and/or immunosuppression becomes even more complicated in COVID-19 patients with a peculiar immunological status, such as pregnancy. 

In fact, even if SARS-CoV-2 infection interferes with all tissues and cells previously mentioned, the immune system is perhaps one of the most involved. In particular, it has been reported that SARS-CoV-2 infection innate immune response modulation could result in both immune hyperactivation or weakening [48]. 

As described by several studies, many cases of COVID-19 are characterized by a decreased innate immune response, with low monocyte levels [49], high neutrophil count [50] and natural killer (NK) cell anergic status [51]. In particular, one of the first-line defenses during viral infection is represented by innate antiviral systems, such RNA-sensors activation, which include different pattern recognition receptors (PRRs), such as RG-I and Toll-like receptors (TLRs). Rizzo et al. demonstrated that specific intracellular TLRs, TLR3 and TLR7, constitute important mediators of anti-viral response during SARS-CoV-2 infection, through the recognition of viral RNA genome. The authors used a Calu-3/MRC-5 3D in vitro lung model, and reported that, after SARS-CoV-2 infection, viral RNA genome recognition by TLR3 and TLR7 led to peculiar responses in terms of production of pro-inflammatory interleukins (ILs) and interferons (IFNs). Precisely, TLR3 engagement was involved in IFN-α and IFN-β production and the secretion of pro-inflammatory cytokines (IL-1 α, IL-1 β, IL-4, IL-6), while TLR7 activation regulates type-1 IFN, IFNγ and IFN-λ3 expression [52] (Figure 1). This study supported the role of these pathways in COVID-19 symptomatology and suggested TLRs as a potential target for new therapies. 

Moreover, besides the activation of innate antiviral systems, such as RNA sensing, the adaptive immune system also plays a central role during SARS-CoV-2 infection. Both humoral and cellular-mediated responses are active mostly against the S1 domain of the SARS-CoV-2 spike protein, with a major activation of CD4+ T cells that support antibody generation too. Antibody responses to SARS-CoV-2, specifically immunoglobulin G (IgG), are fundamental in providing protection against viral infection (Figure 1). Furthermore, the induction of virus-specific neutralizing antibodies within the airways is considered the main immune defense, following natural SARS-CoV-2 infection or vaccination [53]. 

As a matter of fact, a recent study indicates a direct correlation between SARS-CoV-2 neutralizing antibody titer, IgG amount and clinical COVID-19 outcomes. In particular, the study showed that in some subjects, despite having high levels of anti-S1 IgG antibodies, a re-infection may occur. This result indicates that the presence of adequate anti-S1 IgG titers, but not of relevant neutralizing antibodies, represents a possible risk factor for SARS-CoV-2 re-infection [54], supporting the importance of an adequate humoral immune response in SARS-CoV-2 infection resolution.

## Conclusions

Since the occurrence of the new SARS-CoV-2 infection pandemic, more evidence reported that the virus can infect several tissues and organs due to the diffuse expression of SARS-CoV-2 receptors and new entry mechanisms exploited by new SARS-CoV-2 variants.

Nevertheless, even if the respiratory tract remains the main site of SARS-CoV-2 infection, the spectrum of SARS-CoV-2 clinical manifestation is wide [43], and COVID-19 patients experience several complications and adverse manifestation aggravated by the presence of comorbidities, such as diabetes mellitus [23]. 

In this view, the use of both existing therapies and prevention is crucial in decreasing infection rates.

This is also true concerning the reproductive system and particularly pregnancy, where SARS-CoV-2 can take advantage of the peculiar maternal immune system asset, affecting pregnancy outcomes and the fetal microenvironment [47]. In fact, an efficient immune activation is essential to counteract SARS-CoV-2 infection, at both innate and acquired levels. Hence, the continuous monitoring of new variants of SARS-CoV-2 and the increased knowledge of the mechanisms underlying both viral spread strategies and immune response efficiency toward the infection, are fundamental in identifying potential risk factors and developing more efficient strategies for prevention and treatment therapies.

## Figures and Tables

**Figure 1:**
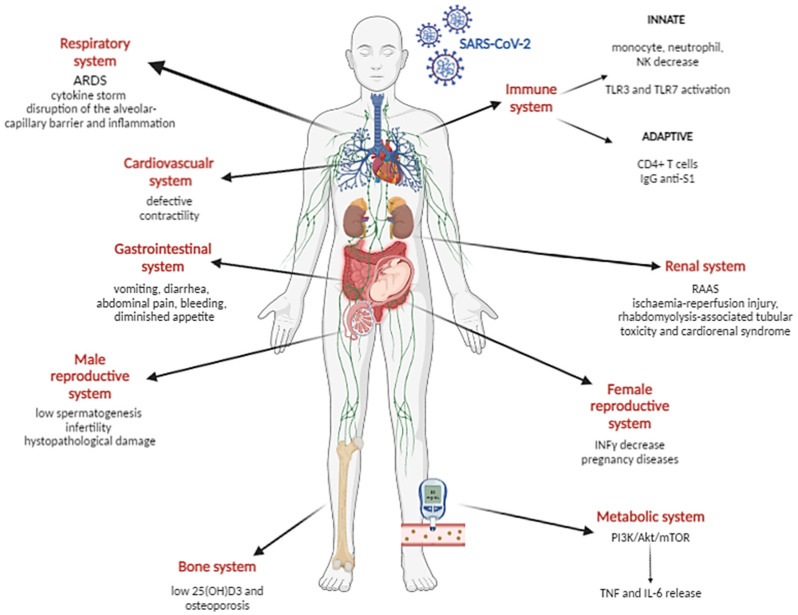
Schematic representation of SARS-CoV-2 infection effects on the immune (innate and adaptive), renal, metabolic, bone, gastrointestinal, cardiovascular, respiratory, bone, female and male reproductive systems.

## References

[1] Kalinina O., Golovkin A., Zaikova E., Aquino A., Bezrukikh V., Melnik O., Vasilieva E., Karonova T., Kudryavtsev I., Shlyakhto E. (2022). Cytokine Storm Signature in Patients with Moderate and Severe COVID-19. Int. J. Mol. Sci..

[2] Guan W.J., Ni Z.Y., Hu Y., Liang W.H., Ou C.Q., He J.X., Liu L., Shan H., Lei C.L., Hui D.S.C. (2020). Clinical Characteristics of Coronavirus Disease 2019 in China. N. Engl. J. Med..

[3] Matthay M.A., Zemans R.L., Zimmerman G.A., Arabi Y.M., Beitler J.R., Mercat A., Herridge M., Randolph A.G., Calfee C.S. (2019). Acute respiratory distress syndrome. Nat. Rev. Dis. Prim..

[4] Caccuri F., Bugatti A., Zani A., De Palma A., Di Silvestre D., Manocha E., Filippini F., Messali S., Chiodelli P., Campisi G. (2021). SARS-CoV-2 Infection Remodels the Phenotype and Promotes Angiogenesis of Primary Human Lung Endothelial Cells. Microorganisms.

[5] Nascimento Conde J., Schutt W.R., Gorbunova E.E., Mackow E.R. (2020). Recombinant ACE2 Expression Is Required for SARS-CoV-2 To Infect Primary Human Endothelial Cells and Induce Inflammatory and Procoagulative Responses. mBio.

[6] Sigrist C.J., Bridge A., Le Mercier P. (2020). A potential role for integrins in host cell entry by SARS-CoV-2. Antivir. Res..

[7] Cao M., Zhang D., Wang Y., Lu Y., Zhu X., Li Y., Xue H., Lin Y., Zhang M., Sun Y. (2020). Clinical Features of Patients Infected with the 2019 Novel Coronavirus (COVID-19) in Shanghai, China. medRxiv.

[8] Spruit M.A., Singh S.J., Garvey C., ZuWallack R., Nici L., Rochester C., Hill K., Holland A.E., Lareau S.C., Man W.D. (2013). An official American Thoracic Society/European Respiratory Society statement: Key concepts and advances in pulmonary rehabilitation. Am. J. Respir. Crit. Care Med..

[9] McCarthy B., Casey D., Devane D., Murphy K., Murphy E., Lacasse Y. (2015). Pulmonary rehabilitation for chronic obstructive pulmonary disease. Cochrane Database Syst. Rev..

[10] Bertolucci F., Sagliocco L., Tolaini M., Posteraro F. (2021). Comprehensive rehabilitation treatment for sub-acute COVID-19 patients: An observational study. Eur. J. Phys. Rehabil. Med..

[11] Spielmanns M., Buelow M.M., Pekacka-Egli A.M., Cecon M., Spielmanns S., Windisch W., Hermann M. (2021). Clinical and Functional Predictors of Response to a Comprehensive Pulmonary Rehabilitation in Severe Post-COVID-19 Patients. Microorganisms.

[12] Albini A., Di Guardo G., Noonan D.M., Lombardo M. (2020). The SARS-CoV-2 receptor, ACE-2, is expressed on many different cell types: Implications for ACE-inhibitor- and angiotensin II receptor blocker-based cardiovascular therapies. Intern. Emerg. Med..

[13] Gupta A., Madhavan M.V., Sehgal K., Nair N., Mahajan S., Sehrawat T.S., Bikdeli B., Ahluwalia N., Ausiello J.C., Wan E.Y. (2020). Extrapulmonary manifestations of COVID-19. Nat. Med..

[14] Lesher A.M., Song W.C. (2010). Review: Complement and its regulatory proteins in kidney diseases. Nephrology.

[15] Izzedine H., Jhaveri K.D. (2021). Acute kidney injury in patients with COVID-19: An update on the pathophysiology. Nephrol. Dial. Transplant..

[16] Nishiga M., Wang D.W., Han Y., Lewis D.B., Wu J.C. (2020). COVID-19 and cardiovascular disease: From basic mechanisms to clinical perspectives. Nat. Rev. Cardiol..

[17] Azevedo R.B., Botelho B.G., Hollanda J.V.G., Ferreira L.V.L., Junqueira de Andrade L.Z., Oei S., Mello T.S., Muxfeldt E.S. (2021). COVID-19 and the cardiovascular system: A comprehensive review. J. Hum. Hypertens..

[18] Soumya R.S., Unni T.G., Raghu K.G. (2021). Impact of COVID-19 on the Cardiovascular System: A Review of Available Reports. Cardiovasc. Drugs Ther..

[19] Ma C., Cong Y., Zhang H. (2020). COVID-19 and the Digestive System. Am. J. Gastroenterol..

[20] Singh A.K., Gupta R., Ghosh A., Misra A. (2020). Diabetes in COVID-19: Prevalence, pathophysiology, prognosis and practical considerations. Diabetes Metab. Syndr..

[21] Di Castelnuovo A., Bonaccio M., Costanzo S., Gialluisi A., Antinori A., Berselli N., Blandi L., Bruno R., Cauda R., Guaraldi G. (2020). Common cardiovascular risk factors and in-hospital mortality in 3,894 patients with COVID-19: Survival analysis and machine learning-based findings from the multicentre Italian CORIST Study. Nutr. Metab. Cardiovasc. Dis..

[22] Mancusi C., Grassi G., Borghi C., Ferri C., Muiesan M.L., Volpe M., Iaccarino G., Group S.-R.I. (2021). Clinical Characteristics and Outcomes of Patients with COVID-19 Infection: The Results of the SARS-RAS Study of the Italian Society of Hypertension. High Blood Press. Cardiovasc. Prev..

[23] Pinchera B., Schiano Moriello N., Buonomo A.R., Di Filippo I., Tanzillo A., Buzzo G., Villari R., Gentile I., Federico Ii Covid T. (2023). Diabetes and SARS-CoV-2 Infection: The Potential Role of Antidiabetic Therapy in the Evolution of COVID-19. Microorganisms.

[24] Cariou B., Hadjadj S., Wargny M., Pichelin M., Al-Salameh A., Allix I., Amadou C., Arnault G., Baudoux F., Bauduceau B. (2020). Phenotypic characteristics and prognosis of inpatients with COVID-19 and diabetes: The CORONADO study. Diabetologia.

[25] Shi Q., Zhang X., Jiang F., Zhang X., Hu N., Bimu C., Feng J., Yan S., Guan Y., Xu D. (2020). Clinical Characteristics and Risk Factors for Mortality of COVID-19 Patients With Diabetes in Wuhan, China: A Two-Center, Retrospective Study. Diabetes Care.

[26] Singh A.K., Gillies C.L., Singh R., Singh A., Chudasama Y., Coles B., Seidu S., Zaccardi F., Davies M.J., Khunti K. (2020). Prevalence of co-morbidities and their association with mortality in patients with COVID-19: A systematic review and meta-analysis. Diabetes Obes. Metab..

[27] Radtke F., MacDonald H.R., Tacchini-Cottier F. (2013). Regulation of innate and adaptive immunity by Notch. Nat. Rev. Immunol..

[28] Cho S.H., Raybuck A.L., Blagih J., Kemboi E., Haase V.H., Jones R.G., Boothby M.R. (2019). Hypoxia-inducible factors in CD4(+) T cells promote metabolism, switch cytokine secretion, and T cell help in humoral immunity. Proc. Natl. Acad. Sci. USA.

[29] Pinchera B., Scotto R., Buonomo A.R., Zappulo E., Stagnaro F., Gallicchio A., Viceconte G., Sardanelli A., Mercinelli S., Villari R. (2022). Diabetes and COVID-19: The potential role of mTOR. Diabetes Res. Clin. Pract..

[30] Walrand S., Guillet C., Boirie Y., Vasson M.P. (2006). Insulin differentially regulates monocyte and polymorphonuclear neutrophil functions in healthy young and elderly humans. J. Clin. Endocrinol. Metab..

[31] Pinchera B., Spirito L., Buonomo A.R., Foggia M., Carrano R., Salemi F., Schettino E., Papa F., La Rocca R., Crocetto F. (2022). mTOR Inhibitor Use Is Associated With a Favorable Outcome of COVID-19 in Patients of Kidney Transplant: Results of a Retrospective Study. Front. Med..

[32] Inoki K., Zhu T., Guan K.L. (2003). TSC2 mediates cellular energy response to control cell growth and survival. Cell.

[33] Musi N., Hirshman M.F., Nygren J., Svanfeldt M., Bavenholm P., Rooyackers O., Zhou G., Williamson J.M., Ljunqvist O., Efendic S. (2002). Metformin increases AMP-activated protein kinase activity in skeletal muscle of subjects with type 2 diabetes. Diabetes.

[34] Hariyanto T.I., Kurniawan A. (2020). Metformin use is associated with reduced mortality rate from coronavirus disease 2019 (COVID-19) infection. Obes. Med..

[35] Isoda K., Young J.L., Zirlik A., MacFarlane L.A., Tsuboi N., Gerdes N., Schonbeck U., Libby P. (2006). Metformin inhibits proinflammatory responses and nuclear factor-kappaB in human vascular wall cells. Arterioscler. Thromb. Vasc. Biol..

[36] Griffin G., Hewison M., Hopkin J., Kenny R.A., Quinton R., Rhodes J., Subramanian S., Thickett D. (2021). Perspective: Vitamin D supplementation prevents rickets and acute respiratory infections when given as daily maintenance but not as intermittent bolus: Implications for COVID-19. Clin. Med..

[37] Bassatne A., Basbous M., Chakhtoura M., El Zein O., Rahme M., El-Hajj Fuleihan G. (2021). The link between COVID-19 and VItamin D (VIVID): A systematic review and meta-analysis. Metabolism.

[38] Teshome A., Adane A., Girma B., Mekonnen Z.A. (2021). The Impact of Vitamin D Level on COVID-19 Infection: Systematic Review and Meta-Analysis. Front. Public Health.

[39] Romero-Ibarguengoitia M.E., Gutiérrez-González D., Cantú-López C., Sanz-Sánchez M.Á., González-Cantú A. (2023). Effect of Vitamin D3 Supplementation vs. Dietary–Hygienic Measures on SARS-CoV-2 Infection Rates in Hospital Workers with 25-Hydroxyvitamin D3 [25(OH)D3] Levels ≥ 20 ng/mL. Microorganisms.

[40] Al-Kuraishy H.M., Al-Gareeb A.I., Faidah H., Al-Maiahy T.J., Cruz-Martins N., Batiha G.E. (2021). The Looming Effects of Estrogen in COVID-19: A Rocky Rollout. Front. Nutr..

[41] Qi J., Zhou Y., Hua J., Zhang L., Bian J., Liu B., Zhao Z., Jin S. (2021). The scRNA-seq Expression Profiling of the Receptor ACE2 and the Cellular Protease TMPRSS2 Reveals Human Organs Susceptible to SARS-CoV-2 Infection. Int. J. Environ. Res. Public Health.

[42] Gye M.C., Kim S.T. (2004). Expression of cathepsin L in human testis under diverse infertility conditions. Arch. Androl..

[43] Louis T.J., Qasem A., Abdelli L.S., Naser S.A. (2022). Extra-Pulmonary Complications in SARS-CoV-2 Infection: A Comprehensive Multi Organ-System Review. Microorganisms.

[44] Campos R.K., Camargos V.N., Azar S.R., Haines C.A., Eyzaguirre E.J., Rossi S.L. (2021). SARS-CoV-2 Infects Hamster Testes. Microorganisms.

[45] Hadjadj J., Yatim N., Barnabei L., Corneau A., Boussier J., Smith N., Pere H., Charbit B., Bondet V., Chenevier-Gobeaux C. (2020). Impaired type I interferon activity and inflammatory responses in severe COVID-19 patients. Science.

[46] Murphy S.P., Tayade C., Ashkar A.A., Hatta K., Zhang J., Croy B.A. (2009). Interferon gamma in successful pregnancies. Biol. Reprod..

[47] Cennamo M., La Civita E., Sarno L., Carbone G., Di Somma S., Cabaro S., Troisi J., Sirico A., Improda F.P., Guida M. (2023). Low Interferon-gamma Levels in Cord and Peripheral Blood of Pregnant Women Infected with SARS-CoV-2. Microorganisms.

[48] Schiuma G., Beltrami S., Bortolotti D., Rizzo S., Rizzo R. (2022). Innate Immune Response in SARS-CoV-2 Infection. Microorganisms.

[49] Rajamanickam A., Kumar N.P., Pandiarajan A.N., Selvaraj N., Munisankar S., Renji R.M., Venkatramani V., Murhekar M., Thangaraj J.W.V., Kumar M.S. (2021). Dynamic alterations in monocyte numbers, subset frequencies and activation markers in acute and convalescent COVID-19 individuals. Sci. Rep..

[50] Meizlish M.L., Pine A.B., Bishai J.D., Goshua G., Nadelmann E.R., Simonov M., Chang C.H., Zhang H., Shallow M., Bahel P. (2021). A neutrophil activation signature predicts critical illness and mortality in COVID-19. Blood Adv..

[51] Bortolotti D., Gentili V., Rizzo S., Rotola A., Rizzo R. (2020). SARS-CoV-2 Spike 1 Protein Controls Natural Killer Cell Activation via the HLA-E/NKG2A Pathway. Cells.

[52] Bortolotti D., Gentili V., Rizzo S., Schiuma G., Beltrami S., Strazzabosco G., Fernandez M., Caccuri F., Caruso A., Rizzo R. (2021). TLR3 and TLR7 RNA Sensor Activation during SARS-CoV-2 Infection. Microorganisms.

[53] Moss P. (2022). The T cell immune response against SARS-CoV-2. Nat. Immunol..

[54] Hocher B., Schonbrunn A., Chen X., Kramer B.K., von Baehr V. (2022). Outliers Matter-Correlation between S1 IgG SARS-CoV-2 Antibodies and Neutralizing SARS-CoV-2 Antibodies. Microorganisms.

